# Prevalence, Characteristics, and Outcomes Associated with Acute Kidney Injury among Adult Patients with Severe Dengue in Mainland China

**DOI:** 10.4269/ajtmh.22-0803

**Published:** 2023-06-26

**Authors:** Changtai Wang, Wenxin Hong, Zhiyue Ou, Huiqin Yang, Lingzhai Zhao, Zhenhua Zhang, Fuchun Zhang

**Affiliations:** ^1^ Department of Infectious Disease, Guangzhou Eighth People’s Hospital, Guangzhou Medical University, Guangzhou, China;; ^2^ Department of Infectious Disease, Guangzhou Red Cross Hospital, Jinan University, Guangzhou, China;; ^3^ Department of Clinical Laboratory, Guangzhou Eighth People’s Hospital, Guangzhou Medical University, Guangzhou, China;; ^4^ Department of Infectious Diseases, The Second Hospital of Anhui Medical University, Hefei, China

## Abstract

Acute kidney injury (AKI) can occur in adult patients with severe dengue (SD) and have serious clinical outcomes. This study aimed to determine the prevalence, characteristics, risk factors, and clinical outcomes of AKI in adult patients with SD; the correlation of dengue virus (DENV) serological and virological profiles with AKI; and the clinical features of patients with severe AKI who received renal replacement treatment (RRT). This multicenter study was conducted in Guangdong Province, China, between January 2013 and November 2019. A total of 242 patients were evaluated, of which 85 (35.1%) developed AKI and 32 (13.2%) developed severe AKI (stage 3). Patients with AKI had a higher fatality rate (22.4% versus 5.7%; *P* < 0.001) and longer length of hospital stay (median: 13 versus 9 days; *P* < 0.001). Independent risk factors for AKI were hypertension (odds ratio [OR]: 2.03; 95% CI: 1.10–3.76), use of nephrotoxic drugs (OR: 1.90; 95% CI: 1.00–3.60), respiratory distress (OR: 4.15; 95% CI: 1.787–9.632), high international normalized ratio (INR) levels (OR: 6.44; 95% CI: 1.89–21.95), and hematuria (OR: 2.12; 95% CI: 1.14–3.95). There was no significant association between DENV serological and virological profiles and the presence or absence of AKI. Among patients with severe AKI, those who received RRT had a longer length of hospital stay and similar fatality rate. Hence, adult patients with SD should be closely monitored for the development of AKI to enable timely and appropriate therapy.

## INTRODUCTION

Dengue fever (DF), caused by the dengue virus (DENV), is one of the most important and rapidly growing viral diseases and poses a severe worldwide public health threat.^1^ The clinical manifestations of DF are diverse, and a small proportion of patients can progress to severe dengue (SD). SD can cause severe damage to vital organs, such as the kidneys, liver, and heart, and even lead to death.^2^^,^^3^

Acute kidney injury (AKI) is a significant and poorly studied complication of dengue, especially in adult patients.^4^^–^^6^ The prevalence of dengue-associated AKI varies from 0.9% to 35.7%, depending on the definition of AKI, age group, and severity.^4^^,^^5^^,^^7^^,^^8^ Dengue-associated AKI is notably observed among hospitalized adult patients with SD, including those with dengue hemorrhagic fever (DHF) or dengue shock syndrome, ranging from 15% to 65%.^5^^,^^9^^–^^12^ Most studies use the 1997 WHO DF case classification,^9^^,^^12^^,^^13^ but this classification does not include organ involvement and may miss some severe cases.^14^ Therefore, it may underestimate the incidence rate of AKI. Furthermore, the results of these studies are inconsistent, and therefore further studies are needed to confirm them.

The pathogenesis and risk factors of AKI in patients with dengue remain unclear.^4^^–^^9^ AKI is associated with increased mortality, prolonged hospitalization, and development of chronic kidney disease (CKD).^15^^,^^16^ Early identification of AKI has the potential to decrease morbidity and mortality.^17^

Owing to the increasing number of SD cases reported in mainland China over the past decade, mainly occurring in adults, further investigation is required to help clinicians prevent AKI by making an accurate early diagnosis.^18^^,^^19^ Although several studies have been conducted in Taiwan, the relevant research is still insufficient, especially in mainland China.^9^^,^^20^ Therefore, this multicenter retrospective study of adult patients with SD was conducted to assess the incidence, clinical characteristics, and risk factors for AKI and its impact on hospital outcomes in mainland China. Furthermore, the correlation of DENV serological and virological profiles with the occurrence of AKI and the clinical features of patients with severe AKI who received renal replacement treatment (RRT) were investigated.

## MATERIALS AND METHODS

### Ethics statement.

This study was conducted in accordance with the principles of the Declaration of Helsinki and was approved by the Ethics Committee of Guangzhou Eighth People’s Hospital, Guangzhou Medical University (no. 20160264). Written informed consent was obtained. Patient data were anonymized prior to analysis.

### Study design.

This multicenter dengue cohort study was performed in 29 hospitals in Guangdong Province, China, which had participated in a previous study.^21^ All patients diagnosed with SD between January 2013 and December 2019 were identified. Patients ≥ 18 years old with SD and a positive dengue nonstructural protein 1 (NS1) antigen result by enzyme-linked immunosorbent assay (ELISA), DENV RNA by real-time polymerase chain reaction (RT-PCR), and/or serum seroconversion of dengue immunoglobulin G (IgG) antibodies on ELISA^3^^,^^22^ who were hospitalized for ≥ 2 days were included in this study. The exclusion criteria were 1) incomplete demographic information, 2) lack of serum creatinine (SCr) levels, 3) time from onset to admission > 7 days, 4) end-stage renal disease receiving RRT, and 5) pregnancy.

### Data collection.

Data on patient demographics and clinical and laboratory findings were collected from patient paper and computerized records and recorded on a structured case record form. Data were also collected on DENV RNA, NS1 antigen, and IgM/IgG antibody test results. Patients who were transferred between hospitals were counted as a single admission. To ensure accuracy, three researchers independently reviewed and checked the data.

Patients at Guangzhou Eighth People’s Hospital, Guangzhou Medical University, had 3–5 mL of blood collected on the first or second day of admission to test for DENV serotypes, viral load, and IgM/IgG antibodies. The DENV serotype and viral load were determined simultaneously using the DENV1-4 One-Step RT-PCR Kit (DaAn Gene, Guangzhou, China) according to the manufacturer’s instructions. Serum IgM and IgG antibodies against DENV were detected using the Dengue Duo IgM and IgG Capture ELISA Kit (Panbio, Brisbane, Australia). The IgM/IgG ratio was used to distinguish between primary and secondary infections. Patients with positive IgM but negative IgG antibodies within 7 days of onset or IgM/IgG ratio ≥ 1.2 were considered to have a primary infection, whereas those with positive IgM and IgG antibodies or only positive IgG antibody within 7 days of onset or IgM/IgG ratio < 1.2 were considered to have a secondary infection.^3^ Each test run included negative and positive controls.

### Definitions.

According to the 2009 WHO and China guidelines for the diagnosis of dengue,^3^^,^^22^ SD was confirmed using at least one of the following criteria: 1) plasma leakage leading to shock and/or respiratory distress; 2) severe bleeding; and 3) severe organ impairment, such as alanine aminotransferase (ALT) and/or aspartate aminotransferase (AST) ≥ 1,000 U/L, myocarditis, encephalopathy, or encephalitis.

AKI was identified and classified on the basis of SCr levels according to Kidney Disease: Improving Global Outcomes (KDIGO) criteria^23^: stage 1, SCr levels increased by 0.3 mg/dL within 48 hours, or 1.5–1.9 times within 7 days from baseline; stage 2, SCr levels increased 2–2.9 times within 7 days from baseline; and stage 3, SCr levels increased 3 times from baseline or by ≥ 4 mg/dL within 7 days. However, because of the lack of urine output data and the use of RRT for early treatment of viral infections, we applied the SCr criteria only. The lowest SCr levels at any time were identified as the patient’s baseline value. In patients with only a single SCr level result, the baseline value was estimated using the simplified modification of diet in renal disease equation by assuming an estimated glomerular filtration rate (eGFR) of 75 mL/minute/1.73 m^2^.^24^ Specifically, eGFR = 186 × (SCr mg/dL)^−1.154^ × age^−0.203^ × 0.742 (if female). Patients were assigned to the AKI group if they developed AKI within 7 days of hospitalization. Patients who developed AKI > 7 days after admission were assigned to the non-AKI group because the occurrence of AKI may have been associated with factors other than DENV infection.

A fatal group was defined as patients who died or discontinued therapy because of clinical deterioration before discharge. Patients with KDIGO stage 3 were defined as having severe AKI. Renal recovery at discharge was classified according to the following criteria: complete recovery, absence of AKI criteria; partial recovery, KDIGO stage improvement but the criteria for AKI disappearance are not met; no recovery, KDIGO stage increased, a need for RRT, or fatality before discharge. The Charlson comorbidity index (CCI) was based on an assessment of survival in the general population^25^ and was calculated from comorbidities extracted from the hospital’s patient records. CKD was considered the underlying illness if the patient had a diagnosis of CKD; other kidney diseases refer to kidney stones and polycystic kidney disease. Nephrotoxic drugs included aminoglycosides, antiviral drugs, vancomycin, antifungal drugs, contrast agents, nonsteroidal anti-inflammatory drugs, and diuretics. The following definitions were used: hematuria was defined as red blood cells ≥ 5 cells per high-power field or dipstick urine blood +2 or above; proteinuria was defined as urinary protein ≥ 30 mg/dL or dipstick urine protein +1 or above; rapid heart rate was defined as heart rate ≥ 100 beats/minute; respiratory distress was defined as respiratory rate ≥ 24 breaths/minute; low systolic blood pressure was defined as < 90 mm of Hg; and low mean arterial pressure was defined as < 65 mm of Hg.

### Statistical analysis.

Categorical variables were expressed as frequencies and percentages, and continuous variables were presented as the median and interquartile range. Categorical variables were analyzed using the χ^2^ test, Fisher’s exact test, or linear-by-linear association test, and continuous variables were analyzed using the Student’s *t* test, Mann–Whitney *U* test, or Kruskal–Wallis test, as appropriate. The risk of fatal outcomes in patients with AKI was evaluated using logistic regression analysis. A stepwise multivariable logistic regression model was used to identify factors associated with AKI. Survival was assessed using the Kaplan–Meier method, and groups were compared using the log-rank test. All statistical analyses were performed using SPSS Statistics version 23 (IBM Corp., Armonk, NY) and Prism version 8 (GraphPad Software Inc., San Diego, CA). All tests were two sided, and statistical significance was set at *P* < 0.05.

## RESULTS

**Figure 1.**
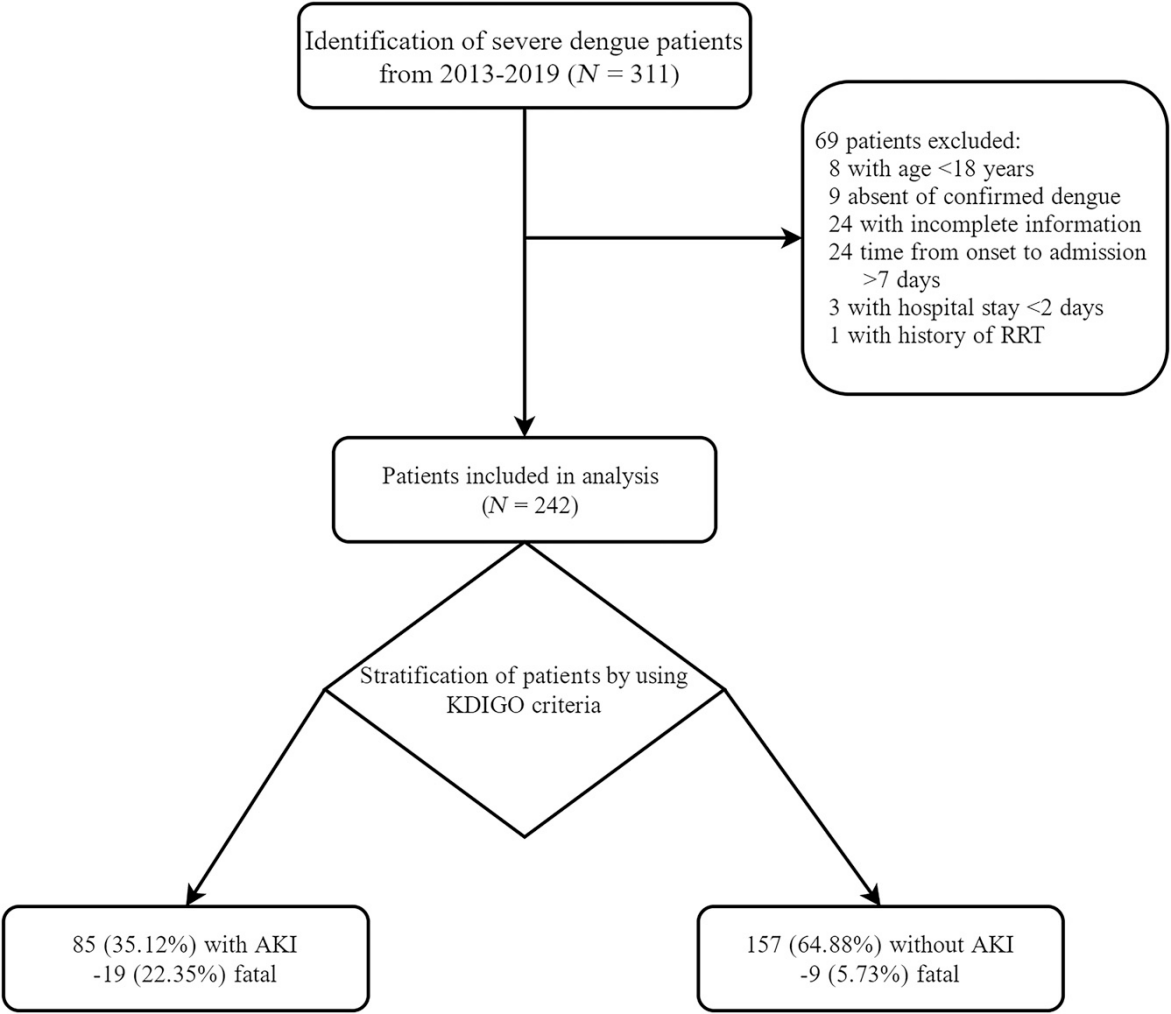
Flowchart of the study. AKI = acute kidney injury; KDIGO = Kidney Disease: Improving Global Outcomes; RRT = renal replacement therapy.

A total of 242 patients were enrolled and eligible for inclusion in the analysis, as shown in Figure 1. According to the KDIGO classification system, 85 (35.1%) patients developed AKI, of which 37 (43.5%), 16 (18.8%), and 32 (37.7%) were classified as having stages 1, 2, and 3, respectively.

The clinical characteristics of SD patients with AKI are shown in Table 1. Patients with AKI had a higher median age than those without AKI (74 versus 70 years); however, the difference was not significant. Patients with AKI had a higher proportion of comorbidities, including hypertension (54.1% versus 38.0%; *P* = 0.022) and diabetes mellitus (28.2% versus 14.0%; *P* = 0.007). The proportion with CCI ≥ 3 in the AKI group was significantly higher than that in the non-AKI group (28.2% versus 15.9%; *P* = 0.023). However, CKD was observed in 17 (20.0%) patients with AKI and 18 (11.5%) without AKI (*P* = 0.072). Table 2 shows patients’ clinical characteristics on admission. The incidence of rapid heart rate, respiratory distress, and pleural effusion or ascites was significantly higher in patients with AKI (*P* < 0.05). Patients with AKI had significantly higher SCr levels (*P* < 0.001) and lower eGFR levels (*P* < 0.001) than those without AKI.

**Table 1:** Characteristics of patients with severe dengue according to acute kidney injury status

Characteristics	All patients (*N* = 242)	Non-AKI patients (*N* = 157)	AKI patients (*N* = 85)	*P *value
Demographics				
Age (years), median (IQR)	70 (51–79)	70 (49–79)	74 (54–80)	0.142
Sex: male, *N* (%)	138 (57.0)	87 (55.4)	51 (60.0)	0.491
Comorbidities, *N* (%)				
CCI ≥ 3	49 (20.2)	25 (15.9)	24 (28.2)	0.023
Hypertension	107 (44.2)	61 (38.9)	46 (54.1)	0.022
Diabetes mellitus	46 (19.0)	22 (14.0)	24 (28.2)	0.007
Cardiovascular diseases	79 (32.6)	54 (34.4)	25 (29.4)	0.430
Previous stroke	45 (18.6)	25 (15.9)	20 (23.5)	0.147
Chronic lung disease	26 (10.7)	14 (8.9)	12 (14.1)	0.212
CKD	35 (14.5)	18 (11.5)	17 (20.0)	0.072
Other kidney disease	23 (9.5)	11 (7.0)	12 (14.1)	0.072
Treatment of SD, *N* (%)				
ACEI or ARB use	29 (12.0)	15 (9.6)	14 (16.5)	0.114
Nephrotoxic drugs	76 (31.4)	41 (26.1)	35 (41.2)	0.016
Human albumin transfusion	125 (51.7)	75 (47.8)	50 (58.8)	0.100
Corticosteroids	45 (18.6)	27 (17.2)	18 (21.2)	0.448
Blood transfusion	121 (50.0)	74 (47.1)	47 (55.3)	0.226
Platelet transfusion	105 (43.4)	67 (42.68)	38 (44.7)	0.761
Red blood cell transfusion	33 (13.6)	14 (8.9)	19 (22.4)	0.004
Plasma transfusion	37 (15.3)	15 (9.6)	22 (25.9)	0.001
Mechanical ventilation	43 (17.8)	16 (10.2)	27 (31.8)	< 0.001
RRT	34 (14.0)	4 (2.5)	30 (35.3)	< 0.001
Clinical outcomes				
Fatality, *N* (%)	28 (11.6)	9 (5.7)	19 (22.4)	< 0.001
Length of hospital stay (days), median (IQR)	10 (6–15)	9 (6–13)	13 (8–24)	< 0.001
Recovery at discharge,* *N* (%)				
Complete recovery	–	–	53 (62.4)	–
Partial recovery	–	–	10 (11.8)	–
No recovery	–	–	22 (25.9)	–

ACEI = angiotensin converting enzyme; AKI = acute kidney injury; ARB = angiotensin receptor blocker; CCI = Charlson comorbidity index; CKD = chronic kidney disease; IQR = interquartile range; RRT = renal replacement therapy; SD = severe dengue.

* Renal recovery at discharge by using the corresponding criterion.

**Table 2:** Clinical characteristics of patients admitted with severe dengue according to acute kidney injury status

Clinical parameters	All patients (*N* = 242)	Non-AKI patients (*N* = 157)	AKI patients (*N* = 85)	*P *value
Rapid heart rate, *N* (%)	45 (18.6)	22 (14.0)	23 (27.1)	0.013
Respiratory distress, *N* (%)	36 (14.9)	11 (7.0)	25 (29.4)	< 0.001
Low SBP, *N* (%)	9 (3.7)	3 (1.9)	6 (7.1)	0.070
Low MAP, *N* (%)	9 (3.7)	4 (2.5)	5 (5.9)	0.284
Pleural effusion or ascites, *N* (%)	72 (29.8)	39 (24.8)	33 (38.8)	0.023
Hepatomegaly, *N* (%)	4 (1.7)	2 (1.3)	2 (2.4)	0.614
WBC (×10^9^/L), median (IQR)	5.2 (3.4–8.1)	4.7 (3.3–7.3)	6.5 (3.7–9.8)	0.005
Hemoglobin (g/L), median (IQR)	125.5 (109–139)	129 (112–140)	122 (100–135)	0.046
Hematocrit (%), median (IQR)	37 (32.7–40.6)	37.5 (33.4–40.9)	36.3 (29.9–39.9)	0.098
PLT (×10^9^/L), median (IQR)	60 (26.8–99.3)	51 (28–97.5)	69 (23.5–125)	0.122
ALT (U/L), median (IQR)	45.5 (27–90.3)	48 (28.5–87.5)	44 (24–101)	0.622
AST (U/L), median (IQR)	83 (46–160)	85 (48–157)	82 (43–197)	0.864
ALB (g/L), median (IQR)	33 (30–36.0)	33.1 (30.2–36.7)	32 (28.9–36)	0.129
SCr (µmol/L), median (IQR)	93.6 (72.0–148.7)	81.9 (67.0–100.6)	161.0 (108.0–342.0)	< 0.001
eGFR (mL/min/1.73 m^2^)	71.4 (37.5–94.4)	82.8 (65.2–109.5)	36.3 (14.4–60.8)	< 0.001
CK (U/L), median (IQR)	301.1 (139–770)	278 (132–609)	410 (160–1273.1)	0.029
LDH (U/L), median (IQR)	380 (285–685)	370 (285–588)	458 (282–978)	0.069
INR, median (IQR)	1.1 (1.0–1.2)	1.0 (1.0–1.1)	1.1 (1.0–1.3)	< 0.001
aPTT (s), median (IQR)	43.2 (36.2–52.4)	43.8 (35.7–50.4)	42 (36.2–53.8)	0.705
Potassium (mmol/L), median (IQR)	3.67 (3.3–4.1)	3.5 (3.3–4.0)	3.8 (3.4–4.2)	0.012
Sodium (mmol/L), median (IQR)	136.8 (134–140)	136.3 (134–139.3)	137.1 (133.3–141)	0.607
Hematuria, *N* (%)	80 (33.1)	43 (27.4)	37 (43.5)	0.011
Proteinuria, *N* (%)	142 (58.7)	89 (56.7)	53 (62.4)	0.393

AKI = acute kidney injury; ALB = albumin; ALT = alanine aminotransferase; aPTT = activated partial thromboplastin time; AST = aspartate aminotransferase; CK = creatine kinase; eGFR = estimated glomerular filtration rate; INR = international normalized ratio; IQR= interquartile range; LDH = lactate dehydrogenase; MAP = mean arterial pressure; PLT = platelet count; SBP = systolic blood pressure; SCr = serum creatinine; WBC = white blood cell count.

Patients with AKI had a higher fatality rate (22.4% versus 5.7%; *P* < 0.001) and longer length of hospital stay (median [interquartile range], 13 [8–24] versus 9 [6–13] days; *P* < 0.001) than those without AKI (Table 1). Among the survivors, 13 patients with AKI did not completely recover, and 2 still required RRT at discharge. An increase in the AKI stage was associated with an increased fatality rate in patients with SD (Supplemental Table 1). Among patients with severe AKI, 12 (37.5%) experienced a complete recovery, 10 (31.3%) had fatal outcomes, and 3 (9.4%) did not recover.

Table 3 shows the results of univariable and multivariable analyses of the risk factors for AKI in patients with SD. Multivariate analysis identified the following independent risk factors for AKI in patients with SD: hypertension (odds ratio [OR]: 2.03; 95% CI: 1.10–3.76; *P* = 0.024), use of nephrotoxic drugs (OR: 1.90; 95% CI: 1.00–3.60; *P* = 0.048), respiratory distress (OR: 4.15; 95% CI: 1.79–9.63; *P* = 0.001), high international normalized ratio (INR) levels (OR: 6.44; 95% CI; 1.89–21.95; *P* = 0.003), and hematuria (OR: 2.12; 95% CI: 1.14–3.95; *P* = 0.018).

**Table 3:** Univariable and multivariable logistic regression to identify risk factors for acute kidney injury in patients with severe dengue

	Univariable analysis		Multivariable analysis	
Variables	OR (95% CI)	*P *value	OR (95% CI)	*P *value
CCI ≥ 3	2.08 (1.10–3.93)	0.024	–	–
Hypertension	1.86 (1.09–3.17)	0.023	2.03 (1.10–3.76)	0.024
Diabetes mellitus	2.41 (1.26–4.64)	0.008	–	–
Nephrotoxic drugs	1.98 (1.13–3.47)	0.017	1.90 (1.00–3.60)	0.048
Red blood cell transfusion	2.94 (1.39–6.22)	0.005	–	–
Plasma transfusion	3.31 (1.61–6.79)	0.001	–	–
Mechanical ventilation	4.10 (2.06–8.18)	< 0.001	–	–
Rapid heart rate	2.28 (1.18–4.39)	0.014	–	–
Respiratory distress	5.53 (2.56–11.95)	< 0.001	4.15 (1.79–9.63)	0.001
Low SBP	3.90 (0.95–16.00)	0.059	–	–
Pleural effusion or ascites	1.92 (1.09–3.39)	0.024	–	–
WBC (×10^9^/L)	1.10 (1.03–1.17)	0.004	–	–
Hemoglobin (g/L)	0.99 (0.98–1.00)	0.043	–	–
CK (U/L)	1.00 (1.00–1.00)	0.061	–	–
INR	9.01 (2.67–30.41)	< 0.001	6.44 (1.89–21.95)	0.003
Potassium (mmol/L)	1.77 (1.13–2.79)	0.013	–	–
Hematuria	2.04 (1.17–3.56)	0.011	2.12 (1.14–3.95)	0.018

CCI = Charlson comorbidity index; CK = creatine kinase; INR = international normalized ratio; OR = odds ratio; SBP = systolic blood pressure; WBC = white blood cell count.

**Figure 2.**
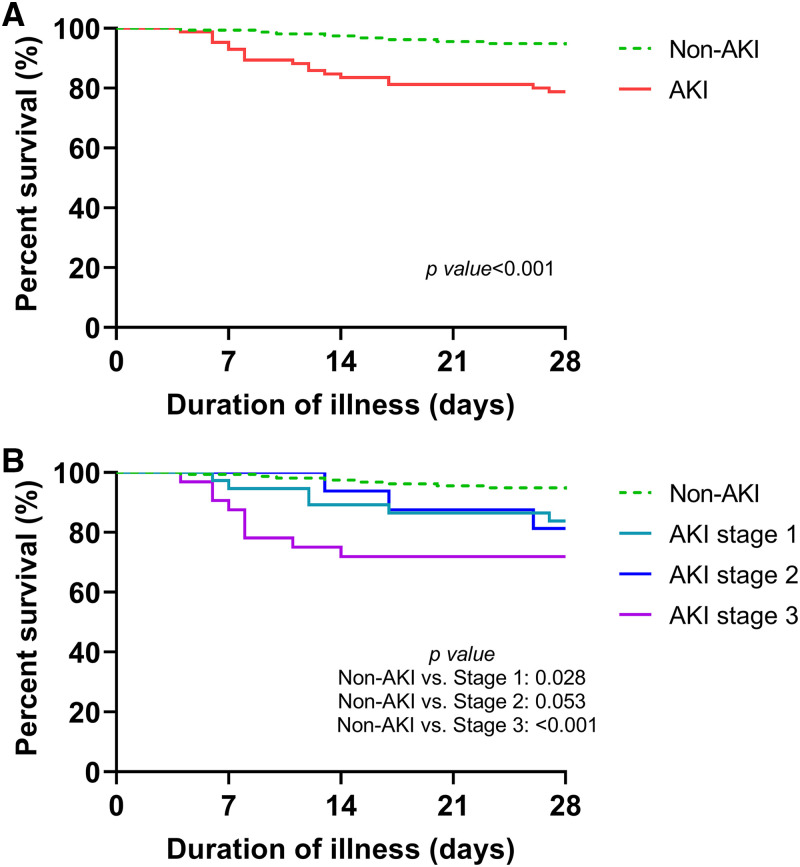
Kaplan–Meier survival curves for the fatality rate among patients with SD. (**A**) With or without AKI. (**B**) Different stages of AKI according to the KDIGO criteria. AKI = acute kidney injury; KDIGO = Kidney Disease: Improving Global Outcomes; SD = severe dengue.

Compared with survivors, patients with fatal outcomes had a higher proportion of those developing AKI (67.9% versus 30.8%; *P* < 0.001) (Table 4). The Kaplan–Meier analysis revealed a significantly higher fatality rate among patients with AKI (*P* < 0.001) and in severe AKI patients (*P* < 0.001) (Figure 2). After adjustment for age, sex, and comorbidity factors in logistic regression models, patients with AKI (OR: 4.34; 95% CI: 1.80–10.47; *P* = 0.001) and severe AKI (OR: 10.77; 95% CI: 3.35–34.57; *P* < 0.001) were at significantly higher risk of fatal outcomes than patients without AKI (Supplemental Table 2). Older age (OR: 1.07; 95% CI: 1.02–1.13; *P* = 0.004), need for mechanical ventilation (OR: 20.61; 95% CI: 6.88–61.71; *P* < 0.001), and need for RRT (OR: 5.18; 95% CI: 1.48–18.15; *P* = 0.010) were independent predictors of fatal outcomes in patients with SD (Table 4).

**Table 4:** Prediction of risk factors for fatal outcomes in patients with severe dengue

Variables	Survivor (*N* = 214)	Fatality (*N* = 28)	*P *value	Multivariable analysis	
				OR (95% CI)	*P *value
Age (years), median (IQR)	69 (49–78)	77 (70–84)	0.001	1.07 (1.02–1.13)	0.004
Sex: male, *N* (%)	121 (56.5)	17 (60.7)	0.675	–	–
CCI ≥ 3, *N* (%)	37 (17.3)	12 (42.9)	0.002	–	–
Pleural effusion or ascites, *N* (%)	60 (28.0)	12 (42.9)	0.107	–	–
Rapid heart rate, *N* (%)	34 (15.9)	11 (39.3)	0.003	–	–
Respiratory distress, *N* (%)	23 (10.7)	13 (46.4)	< 0.001	–	–
Low SBP, *N* (%)	6 (2.8)	3 (10.7)	0.072	–	–
Low MAP, *N* (%)	6 (2.8)	3 (10.7)	0.072	–	–
Human albumin transfusion, *N* (%)	105 (49.1)	20 (71.4)	0.026	–	–
Corticosteroids, *N* (%)	34 (15.89)	11 (39.29)	0.003	–	–
Platelet transfusion, *N* (%)	89 (41.6)	16 (57.1)	0.118	–	–
Red blood cell transfusion, *N* (%)	25 (11.7)	8 (28.6)	0.034	–	–
Plasma transfusion, *N* (%)	25 (11.7)	12 (42.9)	< 0.001	–	–
Mechanical ventilation, *N* (%)	21 (9.8)	22 (78.6)	< 0.001	20.61 (6.88–61.71)	< 0.001
RRT, *N* (%)	21 (9.8)	13 (46.4)	< 0.001	5.18 (1.48–18.15)	0.010
AKI, *N* (%)	66 (30.8)	19 (67.9)	< 0.001	–	–

AKI = acute kidney injury; CCI = Charlson comorbidity index; IQR = interquartile range; MAP = mean arterial pressure; OR = odds ratio; RRT = renal replacement therapy; SBP = systolic blood pressure.

Analysis for evaluation of whether serological (infection type and NS1 antigen) and virological (viral RNA and serotype) profiles were associated with AKI is shown in Table 5. No significant differences in the ratio of secondary dengue infections were observed between patients with and without AKI (28.6% versus 35.4%; *P* = 0.566). The positive rates of DENV NS1 antigen and RNA did not differ significantly between the AKI and non-AKI groups (*P* > 0.05). Among the patients tested, 98 (95.2%) patients were infected with DENV-1 and 5 (4.9%) were infected with DENV-2, with no significant difference between both groups (*P* = 0.134). Furthermore, there were no significant differences in the plasma DENV viral load during the critical phase between AKI and non-AKI patients (median [interquartile range], 3.73 [2.00–5.95] versus 3.97 [2.47–6.73] log_10_ copies/mL; *P* = 0.573).

**Table 5:** Comparison of infection type, NS1 antigen, serotype, and plasma viremia load in patients with severe dengue with and without acute kidney injury

Characteristics	Tested cases	Non-AKI patients	AKI patients	*P *value
Infection type,* *N* (%)				0.566
Primary infection	57 (66.3)	42 (64.6)	15 (71.4)	
Secondary infection	29 (33.7)	23 (35.4)	6 (28.6)	
NS1 antigen,* *N* (%)				0.646
Positive	105 (96.3)	62 (95.4)	43 (97.7)	
Negative	4 (3.7)	3 (4.6)	1 (2.3)	
Serotype,* *N* (%)				0.134
DENV-1	98 (95.1)	72 (97.3)	26 (89.7)	
DENV-2	5 (4.9)	2 (2.7)	3 (10.3)	
Viral RNA, *N* (%)				0.056
Positive	177 (97.3)	116 (99.1)	61 (93.8)	
Negative	5 (2.7)	1 (0.9)	4 (6.2)	
Viral load (3–7 days)*† (log_10_ copies/mL), median (IQR)	3.9 (2.4–6.4)	4.0 (2.5–6.7)	3.7 (2.0–6.0)	0.573

AKI = acute kidney injury; DENV = dengue virus; IQR = interquartile range; NS1 = nonstructural protein 1.

* Data are for patients with dengue recruited from Guangzhou Eighth People’s Hospital.

† Indicates days after the onset of the disease.

Thirty of the 85 patients with AKI (35.3%) and 4 of the 157 patients without AKI (2.5%) received RRT (Table 1). Patients with severe AKI were more likely to receive RRT (21/32; 65.6%) (Supplemental Table 1). Of these, 6 (28.6%) were fatal and 2 (9.5%) still needed RRT after discharge. The fatality rate was similar in patients who did and did not receive RRT (28.6% versus 33.4%; *P* = 0.703). However, patients who received RRT had longer hospital stays than those who did not (median [interquartile range], 25 [7.5–33] versus 6 [3–11] days; *P* = 0.007) (Table 6).

**Table 6:** Characteristics of patients with severe dengue and severe acute kidney injury with and without renal replacement therapy

Parameters	Without RRT (*N* = 11)	With RRT (*N* = 21)	*P *value
Age (years), median (IQR)	74 (57–82)	54 (49–73)	0.084
Sex: male, *N* (%)	5 (45.5)	13 (61.9)	0.465
CCI ≥ 3, *N* (%)	6 (54.5)	4 (19.0)	0.056
Time from onset to admission (days), median (IQR)	4 (3–7)	5 (3.5–6)	0.451
Time from onset to AKI (days)	5 (3–7)	5 (4.5–6.5)	0.871
Mechanical ventilation, *N* (%)	4 (36.4)	9 (42.9)	> 0.999
Fatality, *N* (%)	4 (36.4)	6 (28.6)	0.703
Length of hospital stay (days), median (IQR)	6 (3–11)	25 (7.5–33)	0.007
Recovery at discharge, *N* (%)			
Complete recovery	3 (27.3)	9 (42.9)	–
Partial recovery	3 (27.3)	4 (19.0)	–
No recovery	5 (45.5)	8 (38.1)	–

AKI = acute kidney injury; CCI = Charlson comorbidity index; IQR = interquartile range; RRT = renal replacement therapy.

## DISCUSSION

In this study, approximately one-third of patients with SD developed AKI, which was associated with a higher fatality rate and longer hospital stay. In addition, hypertension, use of nephrotoxic drugs, respiratory distress, high INR levels, and hematuria were independently associated with the development of AKI. This is a relatively large and comprehensive report on SD-associated AKI in adult patients from nonendemic countries. These findings highlight the urgent need to increase awareness and improve patient care, including early diagnosis, timely recognition of clinical deterioration, and prevention of renal complications.

This multicenter retrospective study assessed the association of AKI with patients with SD. Most previous studies used the risk, injury, failure, loss, and end-stage kidney disease (RIFLE) and Acute Kidney Injury Network (AKIN) criteria to assess AKI^4^^,^^12^^,^^26^; however, the KDIGO criteria are more sensitive than the RIFLE and AKIN criteria.^27^^,^^28^ Several studies used admission SCr as the baseline SCr, which may underestimate the severity of disease in the patient.^11^ According to prior reports, SD-associated AKI usually occurs in the early stages.^4^^,^^11^ In comparison to previous studies,^5^^,^^29^^–^^31^ this study evaluated AKI within 7 days of admission, used KDIGO SCr criteria alone, and used the lowest SCr as the baseline SCr, thus allowing for better evaluation.

The reported incidence of AKI in SD patients has varied widely.^4^^,^^5^^,^^11^^,^^12^ Mallhi et al.^26^ reported that the incidence and risk factors of dengue-associated AKI varied according to the diagnostic criteria. In this study, a relatively high incidence of AKI, according to the KDIGO criteria, was observed in adult patients with SD (35.1%), compared with previous studies of adults in Vietnam (15.0%) and Thailand (21.1%).^5^^,^^11^ However, the variations can be partially explained by variable study populations and inconsistent use of AKI criteria. Nevertheless, this study found that the incidence of AKI was high, even if only SCr levels were used as an indicator, which may have underestimated the incidence and severity of AKI in patients.

In this study, the fatality rate of patients with SD was 11.6%, whereas that of patients with AKI was 22.4%. Patients with AKI had a higher risk of fatal outcomes than patients without AKI. The Kaplan–Meier survival curve analysis showed a significant difference in survival between the AKI and non-AKI group, with patients with severe AKI having a higher fatality rate. The need for RRT is a risk factor for fatal outcomes in patients with SD. These results are consistent with previous studies.^5^^,^^11^ Because patients with dengue who survive AKI are at risk of CKD, long-term follow-up is warranted.^32^ SD-associated AKI is not uncommon and is an extremely harmful complication, and its importance has often been neglected during the management of patients with SD.^4^^,^^6^

The pathogenesis of dengue-associated AKI is unclear and may be complex, caused by the direct action of the virus, immune mechanisms, hemolysis, rhabdomyolysis, or shock.^33^ One of the most controversial mechanisms is the direct action of viruses. Some studies have detected viral antigens or loads in the renal tissue pathology of patients with DENV infection.^34^^,^^35^ In this study, no significant association was observed between the serological and virological profile characteristics of DENV and the presence or absence of AKI. Therefore, direct dengue viral invasion may not be the primary cause of AKI in patients with dengue infections, even if it does play a role in damaging renal function. This study also reviewed other possible mechanisms, including hemodynamic instability, shock, and rhabdomyolysis. Patients with SD develop complex diseases, and there is little evidence to support a single mechanism causing AKI, because two or more mechanisms are usually involved.

In this study, several comorbidities were the likely risk factors for AKI. In a study from Taiwan, acute renal failure in patients with DHF predominantly occurred in older men and those who had comorbidities.^9^ Hypertension was the most frequently observed comorbidity in patients with AKI, which is an important cause of microvasculitis and macrovasculitis, which predispose patients to AKI. Although this study did not identify diabetes as a risk factor for AKI, in contrast to a study from Thailand,^11^ this cannot be ruled out because early diabetes may not have been diagnosed or may not be the main factor; further investigation of diabetes as a risk factor is needed. About 40% of patients with AKI use nephrotoxic drugs, which is a risk factor associated with AKI, consistent with previous studies.^10^^,^^26^ Exposure to potentially nephrotoxic drugs should be modified or tailored to reduce the risk of AKI among patients with SD. Patients with respiratory distress also tended to develop AKI in this study, similar to data on patients with coronavirus disease 2019.^36^ In this study, patients with AKI had a prolonged INR in addition to thrombocytopenia, which suggests potential development of disseminated intravascular coagulation. In addition, hematuria was a risk factor associated with AKI. Therefore, in patients with hypertension accompanied by shock, coagulation dysfunction, respiratory distress, hematuria, or the use of nephrotoxic drugs, it is necessary to be alert to the occurrence and development of AKI.

Some predictive models based on logistic regression or machine-learning models have been developed for predicting the development of AKI among patients with sepsis or critical illness.^37^^–^^39^ This study found that the use of nephrotoxic drugs, respiratory distress, hematuria, and high INR levels are independent risk factors for AKI in adult patients with SD. However, these results need to be validated by prospective studies and used to develop predictive models for early assessment of SD-associated AKI.

Apart from the need for RRT, age and the need for mechanical ventilation were independent predictors of fatal outcomes in patients with SD. Previous studies have confirmed that the risk of death from dengue increases with age.^40^^–^^42^ A large study reported that older patients with dengue were at markedly increased risk of death in Singapore.^40^ A recent study involving patients with malaria, dengue, and leptospirosis showed that the need for mechanical ventilation and dialysis was a highly predictive factor for 30-day mortality.^43^ Most patients who need mechanical ventilation and RRT have more serious complications, which should be paid more attention.

In addition to adequate fluid resuscitation and avoidance of nephrotoxic drugs, there is no specific prevention strategy for dengue-associated AKI.^10^ The role of RRT in treating diseases is expanding because it is an important method for eliminating toxins from the body, maintaining and replacing the function of vital organs, and reducing inflammatory storms and body fluids.^44^ The need for RRT varies from 0% to 25% of patients with dengue.^11^^,^^12^^,^^45^^,^^46^ In this study, RRT was required by 14.1% of all patients, 35.3% of patients with AKI, and 65.6% of patients with severe AKI. This proportion is higher than in previous reports,^11^^,^^12^ possibly because this study included critically ill patients and different treatment strategies are used in mainland China.^22^ The fatality rate among patients receiving RRT was 28.6%, and two patients remained RRT dependent. In addition, patients who received RRT had longer hospital stays. However, RRT is a temporary intervention and is associated with a short-term poor prognosis rate similar to that in patients with severe AKI who did not receive RRT. Patients with SD often have thrombocytopenia, and studies have shown that patients with decreased platelet counts receiving continuous RRT are associated with an increased risk of secondary infection.^47^ The efficacy of RRT for SD-associated AKI remains unclear, and there is no consensus on key issues, such as RRT method selection, dose, and timing, which need to be explored in clinical practice.

This study has some limitations. First, this was a multicenter retrospective observational study, and the diagnosis and treatment depended on clinicians’ decisions; therefore, the possibility of bias cannot be excluded. Second, the number of patients with SD was relatively small, so some risk factors might be underestimated because of their rare occurrence. Third, because the majority of patients identified in this study were infected with DENV-1, the conclusion primarily comes from DENV-1-infected individuals. Fourth, some possible confounding factors, such as secondary bacterial infection and the type of fluid resuscitation, might have been missed. Finally, there was no follow-up on the renal function of patients after discharge.

## CONCLUSION

This study found that approximately one-third of adult patients with SD eventually develop AKI and have a poorer prognosis. Hypertension, use of nephrotoxic drugs, respiratory distress, hematuria, and high INR levels were the most significant factors associated with AKI in adult patients with SD. Furthermore, several patients with AKI do not return to normal after discharge and may still require RRT. This study’s findings provide robust evidence that adult patients with SD should be closely monitored in view of the development of AKI, which will help clinicians initiate timely and appropriate therapy for SD. A prospective study on the clinical characteristics and predictive modeling of early AKI in patients with SD will be conducted using the KDIGO criteria in future research.

## Supplemental Material


Supplemental materials

## References

[1] Bhatt S , 2013. The global distribution and burden of dengue. Nature 496: 504–507. 23563266 10.1038/nature12060 PMC3651993

[2] Wilder-Smith A Ooi EE Horstick O Wills B, 2019. Dengue. Lancet 393: 350–363. 30696575 10.1016/S0140-6736(18)32560-1

[3] World Health Organization , 2009. Dengue: Guidelines for Diagnosis, Treatment, Prevention and Control, new edition. Geneva, Switzerland: WHO. 23762963

[4] Surasombatpattana S Sangthawan P Hortiwakul T Charoenmak B Chusri S, 2021. Characteristics and outcomes of adults hospitalized with dengue viral infection and acute kidney injury in southern Thailand. Am J Trop Med Hyg 105: 425–434. 34125698 10.4269/ajtmh.21-0130 PMC8437172

[5] Huy BV Thuy DT, 2020. Prevalence, characteristics, and factors associated with acute kidney injury among adult dengue patients in Vietnam. Am J Trop Med Hyg 104: 1067–1071. 33319734 10.4269/ajtmh.20-0840 PMC7941826

[6] Diptyanusa A Phumratanaprapin W, 2021. Predictors and outcomes of dengue-associated acute kidney injury. Am J Trop Med Hyg 105: 24–30. 33939642 10.4269/ajtmh.21-0007 PMC8274771

[7] Laoprasopwattana KM Pruekprasert PM Dissaneewate PM Geater AP Vachvanichsanong PM, 2010. Outcome of dengue hemorrhagic fever-caused acute kidney injury in Thai children. J Pediatr 157: 303–309. 20362302 10.1016/j.jpeds.2010.02.008

[8] Basu G Chrispal A Boorugu H Gopinath KG Chandy S Prakash JA Thomas K Abraham AM John GT, 2011. Acute kidney injury in tropical acute febrile illness in a tertiary care centre—RIFLE criteria validation. Nephrol Dial Transplant 26: 524–531. 20702532 10.1093/ndt/gfq477

[9] Lee IK Liu JW Yang KD, 2009. Clinical characteristics, risk factors, and outcomes in adults experiencing dengue hemorrhagic fever complicated with acute renal failure. Am J Trop Med Hyg 80: 651–655. 19346394

[10] Mallhi TH Khan AH Adnan AS Sarriff A Khan YH Jummaat F, 2015. Incidence, characteristics and risk factors of acute kidney injury among dengue patients: a retrospective analysis. PLoS One 10: e0138465. 26421839 10.1371/journal.pone.0138465 PMC4589349

[11] Diptyanusa A Phumratanaprapin W Phonrat B Poovorawan K Hanboonkunupakarn B Sriboonvorakul N Thisyakorn U, 2019. Characteristics and associated factors of acute kidney injury among adult dengue patients: a retrospective single-center study. PLoS One 14: e0210360. 30615667 10.1371/journal.pone.0210360 PMC6322747

[12] Patel M Himanshu D Chaudhary S Atam V Sachan R Misra R Mohapatra S, 2019. Clinical characteristic and risk factors of acute kidney injury among dengue viral infections in adults: a retrospective analysis. Indian J Nephrol 29: 15–21. 30814788 10.4103/ijn.IJN_437_17 PMC6375009

[13] Eswarappa M Reddy SB John MM Suryadevara S Madhyashatha RP, 2019. Renal manifestations of dengue viral infection. Saudi J Kidney Dis Transpl 30: 394–400. 31031376 10.4103/1319-2442.256847

[14] Dussart P , 2020. Comparison of dengue case classification schemes and evaluation of biological changes in different dengue clinical patterns in a longitudinal follow-up of hospitalized children in Cambodia. PLoS Negl Trop Dis 14: e0008603. 32925941 10.1371/journal.pntd.0008603 PMC7515206

[15] Chertow GM Burdick E Honour M Bonventre JV Bates DW, 2005. Acute kidney injury, mortality, length of stay, and costs in hospitalized patients. J Am Soc Nephrol 16: 3365–3370. 16177006 10.1681/ASN.2004090740

[16] Coca SG Singanamala S Parikh CR, 2012. Chronic kidney disease after acute kidney injury: a systematic review and meta-analysis. Kidney Int 81: 442–448. 22113526 10.1038/ki.2011.379 PMC3788581

[17] Kaddourah A Basu RK Bagshaw SM Goldstein SL, 2017. Epidemiology of acute kidney injury in critically ill children and young adults. N Engl J Med 376: 11–20. 27959707 10.1056/NEJMoa1611391 PMC5322803

[18] Li D , 2023. The whole-genome sequencing of prevalent DENV-1 strains during the largest dengue virus outbreak in Xishuangbanna Dai Autonomous Prefecture in 2019. J Med Virol 95: e28115. 36059257 10.1002/jmv.28115

[19] Wu W Ren H Lu L, 2021. Increasingly expanded future risk of dengue fever in the Pearl River Delta, China. PLoS Negl Trop Dis 15: e0009745. 34559817 10.1371/journal.pntd.0009745 PMC8462684

[20] Chang K , 2017. Differences in mortality and clinical manifestations of dengue hemorrhagic fever in Taiwan in different years: a comparison for cases in 2014 and 2015 epidemics. Am J Trop Med Hyg 97: 361–368. 28722609 10.4269/ajtmh.16-1018 PMC5544090

[21] Hong HW , 2022. Clinical features of adult patients with severe dengue in Guangdong Province from 2013 to 2019. Chin J Infect Dis 40: 13–19.

[22] Zhang FC , 2011. Guidelines for the diagnosis and treatment of dengue in China. Infect Dis Immun 1: 144–152.

[23] Khwaja A, 2012. KDIGO clinical practice guidelines for acute kidney injury. Nephron Clin Pract 120: c179–c184. 22890468 10.1159/000339789

[24] National Kidney Foundation , 2002. K/DOQI clinical practice guidelines for chronic kidney disease: evaluation classification stratification. Am J Kidney Dis 39: S1–S266. 11904577

[25] Charlson ME Pompei P Ales KL MacKenzie CR, 1987. A new method of classifying prognostic comorbidity in longitudinal studies: development and validation. J Chronic Dis 40: 373–383. 3558716 10.1016/0021-9681(87)90171-8

[26] Mallhi TH Khan AH Sarriff A Adnan AS Khan YH Jummaat F, 2016. Defining acute kidney injury in dengue viral infection by conventional and novel classification systems (AKIN and RIFLE): a comparative analysis. Postgrad Med J 92: 78–86. 26729887 10.1136/postgradmedj-2015-133582

[27] Er RE Okyay GU Kmaz B Türko GA Lu M Erten Y, 2020. Comparison between RIFLE, AKIN, and KDIGO: acute kidney injury definition criteria for prediction of in-hospital mortality in critically ill patients. Iran J Kidney Dis 14: 365–372. 32943591

[28] Tsai TY Chien H Tsai FC Pan HC Yang HY Lee SY Hsu HH Fang JT Yang CW Chen YC, 2017. Comparison of RIFLE, AKIN, and KDIGO classifications for assessing prognosis of patients on extracorporeal membrane oxygenation. J Formos Med Assoc 116: 844–851. 28874330 10.1016/j.jfma.2017.08.004

[29] Jung JY , 2011. Acute kidney injury in critically ill patients with pandemic influenza A pneumonia 2009 in Korea: a multicenter study. J Crit Care 26: 577–585. 21489748 10.1016/j.jcrc.2011.02.012

[30] Argyropoulos A Townley S Upton PM Dickinson S Pollard AS, 2019. Identifying on admission patients likely to develop acute kidney injury in hospital. BMC Nephrol 20: 56. 30764796 10.1186/s12882-019-1237-x PMC6376785

[31] Mekangkul E Siripen N Rianthavorn P, 2023. Prevalence and risk factors of acute kidney injury in hospitalized children with dengue infection using Kidney Disease Improving Global Outcomes criteria. Indian J Pediatr 90: 525. 36943633 10.1007/s12098-023-04524-w

[32] Mallhi TH Khan AH Adnan AS Sarriff A Khan YH Gan SH, 2018. Short-term renal outcomes following acute kidney injury among dengue patients: a follow-up analysis from large prospective cohort. PLoS One 13: e0192510. 29481564 10.1371/journal.pone.0192510 PMC5826532

[33] Christopher TSL Fuah KW Lee SE Kaniappan KK Then RF, 2019. Dengue-associated acute kidney infection: an updated and comprehensive qualitative review of literature. EMJ Nephrol 7: 86–94.

[34] Nunes PCG , 2019. Renal injury in DENV-4 fatal cases: viremia, immune response and cytokine profile. Pathogens 8: 223. 31703246 10.3390/pathogens8040223 PMC6963280

[35] Jessie K Fong MY Devi S Lam SK Wong KT, 2004. Localization of dengue virus in naturally infected human tissues, by immunohistochemistry and in situ hybridization. J Infect Dis 189: 1411–1418. 15073678 10.1086/383043

[36] Kolhe NV Fluck RJ Selby NM Taal MW Remuzzi G, 2020. Acute kidney injury associated with COVID-19: a retrospective cohort study. PLoS Med 17: e1003406. 33125416 10.1371/journal.pmed.1003406 PMC7598516

[37] Yue S Li S Huang X Liu J Hou X Zhao Y Niu D Wang Y Tan W Wu J, 2022. Machine learning for the prediction of acute kidney injury in patients with sepsis. J Transl Med 20: 215. 35562803 10.1186/s12967-022-03364-0 PMC9101823

[38] Fan C Ding X Song Y, 2021. A new prediction model for acute kidney injury in patients with sepsis. Ann Palliat Med 10: 1772–1778. 33353355 10.21037/apm-20-1117

[39] Palomba H Cubos D Bozza F Zampieri FG Romano TG, 2023. Development of a risk score for AKI onset in COVID-19 patients: COV-AKI score. BMC Nephrol 24: 46. 36859175 10.1186/s12882-023-03095-4 PMC9977632

[40] Rowe EK Leo YS Wong JG Thein TL Gan VC Lee LK Lye DC, 2014. Challenges in dengue fever in the elderly: atypical presentation and risk of severe dengue and hospital-acquired infection. PLoS Negl Trop Dis 8: e2777. 24699282 10.1371/journal.pntd.0002777 PMC3974675

[41] Woon YL Hor CP Hussin N Zakaria A Goh PP Cheah WK, 2016. A two-year review on epidemiology and clinical characteristics of dengue deaths in Malaysia, 2013–2014. PLoS Negl Trop Dis 10: e0004575. 27203726 10.1371/journal.pntd.0004575 PMC4874788

[42] Santana LMR Baquero OS Maeda AY Nogueira JS Chiaravalloti Neto F, 2022. Spatio-temporal dynamics of dengue-related deaths and associated factors. Rev Inst Med Trop São Paulo 64: e30. 35384961 10.1590/S1678-9946202264030 PMC8993154

[43] Prabhu MV Arun S Ramesh V, 2016. Fever, thrombocytopenia, and AKI-A profile of malaria, dengue, and leptospirosis with renal failure in a South Indian tertiary-care hospital. Clin Nephrol 86: 128–130. 27509584 10.5414/CNP86S118

[44] Sutherland SM Kwiatkowski DM, 2017. Acute kidney injury in children. Adv Chronic Kidney Dis 24: 380–387. 29229169 10.1053/j.ackd.2017.09.007

[45] Vakrani GP Subramanyam NT, 2017. Acute renal failure in dengue infection. J Clin Diagn Res 11: OC10–OC13. 10.7860/JCDR/2017/22800.9289 PMC5324436 28273991

[46] Padyana M Karanth S Vaidya S Gopaldas JA, 2019. Clinical profile and outcome of dengue fever in multidisciplinary intensive care unit of a tertiary level hospital in India. Indian J Crit Care Med 23: 270–273. 31435145 10.5005/jp-journals-10071-23178 PMC6698353

[47] Griffin BR Wu C O’Horo JC Faubel S Jalal D Kashani K, 2021. The association of platelet decrease following continuous renal replacement therapy initiation and increased rates of secondary infections. Crit Care Med 49: e130–e139. 33372743 10.1097/CCM.0000000000004763 PMC8530244

