# Supplementary material for: Prevalence, Characteristics, and Outcomes Associated with Acute Kidney Injury among Adult Patients with Severe Dengue in Mainland China
Source: Am J Trop Med Hyg. 2023 Jun 26;109(2):404–12. doi: 10.4269/ajtmh.22-0803 (PMC10397446; doi:10.4269/ajtmh.22-0803)
Supplement: Supplementary file 1 [file tpmd220803.SD1.pdf]

TABLE S1 Association between different stages of AKI and outcomes in patients with severe dengue

Outcomes	Non–AKI	Stage 1	Stage 2	Stage 3	*P*–value* all	*P*–value** 0 versus 1 stage
N (%)	157 (64.9)	37 (15.3)	16 (6.6)	32 (13.2)		
RRT, N (%)	4 (2.5)	9 (24.3)	0 (0.0)	21 (65.6)	<0.001	<0.001
Mechanical ventilation, N (%)	16 (10.2)	10 (27.0)	4 (25.0)	13 (40.6)	<0.001	0.013
Fatality, N (%)	9 (5.7)	6 (16.2)	3 (18.8)	10 (31.3)	<0.001	0.043
Length of hospital stay (days), median (IQR)	9 (6–13)	13 (9–20)	14.5 (11–25.5)	11 (5–28)	<0.001	0.002
Recovery at discharge, N (%)						
Complete recovery	–	28 (75.7)	13 (81.3)	12 (37.5)	–	–
Partial recovery	–	3 (8.1)	0 (0.0)	7 (21.9)	–	–
No recovery	–	6 (16.2)	3 (18.8)	13 (40.6)	–	–

AKI = acute kidney injury; RRT = renal replacement therapy.

* *P*-value denotes the trend of association through all AKI stages, by linear–by–linear association test for categorical variables and Kruskal–Wallis test for continuous variables; ** *P*-value denotes the difference between no AKI and Stage 1 AKI, by Chi square test for categorical variables and Wilcoxon rank sums test for continuous variables.

TABLE S2 Risk of fatal outcomes associated with acute kidney injury in logistic regression models

AKI stage	Unadjusted		Adjusted[a]	
	Odds Ratio (95% CI)	*P*–value	Odds Ratio (95% CI)	*P*–value
Non-AKI	1		1	
Stage 1	3.18 (1.06–9.59)	0.040	2.56 (0.82–8.04)	0.106
Stage 2	3.80 (0.91–15.77)	0.066	2.88 (0.64–12.90)	0.168
Stage 3	7.48 (2.73–20.44)	<0.001	10.77 (3.35–34.57)	<0.001
Stage 1–2	3.36 (1.26–8.99)	0.016	2.66 (0.96–7.37)	0.060
Stage 2–3	6.11 (2.42–15.42)	<0.001	6.61 (2.40–18.23)	<0.001
Any AKI	4.73 (2.04–11.02)	<0.001	4.34 (1.80–10.47)	0.001

AKI = acute kidney injury; CI = confidence interval.

[a]Other factors enrolled in multivariate logistic regression model included age, sex, and Charlson's comorbidity score ≥ 3.
